# The Book World of Medicine and Science

**Published:** 1894-03-10

**Authors:** 


### THE BOOK WORLD OF MEDICINE AND SCIENCE.

### BOOK REVIEWS.

THE OPHTHALMOSCOPE: A Manual for Students. By GUSTAVUS HARTRIDGE, F.R.C.S., Surgeon to the Royal Westminster Ophthalmic Hospital, &c. With sixty-seven illustrations and four plates. Second edition. Small 8vo., pp. 156. Price 4s. 6d. (London: J. and A. Churchill, 1894.)

That a book on the ophthalmoscope has reached a second edition may be taken to afford evidence that it has supplied a demand; and Mr. Hartridge is entitled to the credit of having described, with clearness and accuracy, the principles on which the different forms of the instrument are founded, and the manner in which these forms should be used. He has also given a brief account of the structures which the ophthalmoscope displays, and of some of the morbid conditions to which they are liable. We rather doubt the educational value of little books on bits of great subjects, and entertain a suspicion that they are conducive to cramming rather than to study; but this, after all, is a question for the purchaser. We have heard, moreover, that some of the numerous spectacle sellers who, in despite of the Johnsonian definition, describe themselves as "opticians," have of late adopted some pretence of using an ophthalmoscope upon their customers, which is much as if a tailor were to take to the employment of a stethoscope. Mr. Hartridge's treatise is more likely, we think, to appeal to the spurious optician than to the genuine medical student, whose requirements in many particulars, both pathological and therapeutical, it would be inadequate to satisfy. Generally, as we have said, the book is fairly correct, but we cannot refrain from calling attention to the curious error contained in the following passage (p. 85): "The macula (lutea) is never yellow, and it is rather unfortunate that it should have received the name of the yellow spot: this colour is only found as a post-mortem change." Now the truth is that the yellow of the macula cannot be seen in the living eye with the ophthalmoscope on account of the orange or red background on which it rests, and that it is conspicuous after death because the retina becomes whitish and opaque, and the yellow tint is then very visible against the surrounding whiteness. But the yellow of the macula can be demonstrated during life in many ways. It is well known to astronomers that it diminishes the distinctness of faint stars, which are better seen by allowing their images to fall upon the retina just outside the macula; and the variations in the tone and depth of the retinal yellow, which have been noted by many observers, seem to furnish the only adequate explanation of a fact to which Lord Rayleigh was the first to call attention, namely, that different persons, all of them of normal colour-vision in every other respect, will require mixtures of different proportions of the red and the green of the spectrum, in order to obtain what seems to them a perfect match for a given yellow. Sir George Stokes, again, devised and has frequently exhibited an ingenious experiment by which the yellow of the macula may be rendered evident to its possessor. If the beam of an electric lamp be suffered to pass through a cell containing a solution of chloride of chromium, and then to fall upon a white screen, the green colour of the solution will be more absorbed by the yellow spot than by the surrounding portions of the retina, and the result will be that those who look at the white surface will see a faint roseate cloud floating in the centre of the field. The descriptions of this appearance given by different persons fully confirm the belief that the colour of the yellow spot is more pronounced in some individuals than in others, and hence that the cloud is more conspicuous to the former than to the latter, but it is more or less visible to all. Mr. Hartridge will therefore do well to reconsider his teaching, at least in this particular.

THE GREAT PESTILENCE. By FRANCIS AIDAN GASQUET, D.D., O.S.B. (London: Simkin, Marshall, Hamilton, Kent, and Co. 1893.)

Specialists in all matters are liable to the charge of concentrating too close an observation on their own particular subject, and Dr. Francis Gasquet cannot be named among the exceptions which go to prove this rule. The writer combines in his book a strict historical accuracy as to detail with a happy knack of imparting information, but too much stress is laid, we think, on the importance of "The Great Pestilence" as the turning point in the national life. "It formed the real close," in the author's own words, "of the mediæval period and the beginning of our modern age . . . it was the dawn of a new era"; and he declares further on that the Black Death by the sweeping away of the population and the consequent scarcity of labourers raised extravagant expectations in the minds of the lower classes. "Labour then began to understand its value and to assert its power," he says. The ravages of the terrible pestilence did undoubtedly bring about many social and political changes, but it is to be questioned whether they were of sufficiently radical a nature to warrant the author's decided views on the subject. What are of especial interest in Dr. Gasquet's book, from a medical point of view, are the accounts of the various attempts made to arrest the spread of this scourge which visited Europe in the fourteenth century. We are all cognisant of the fact that the Black Death swept off half the population, but we are less well informed as to how far measures were taken to avert the spread of this fell destroyer. Dr. Gasquet furnishes us with much contemporary testimony as to the fatal effects of the disease on the stricken, but the actual nature of the Black Death is not revealed. Whilst the plague was at its height, King Philip VI. requested the medical faculty to consult together and report upon the best methods by which the deadly hold of the disease could be combated, we are informed on the author's authority; unhappily their reply does not contain any historical details. This "commission" thus authorised by the Sovereign do not appear to have thrown much light on the object of their investigations, though they seem to have been pretty clear as to the infectious nature of the disease, for which reason total desertion of the afflicted was prescribed! Indeed, the sick appear to have had neither medical attendants, nurses, nor undertakers; the clergy alone ministered to their needs. Dr. Gasquet frequently calls our attention to their devotion to the miserable sufferers, which fact he authentically substantiates, but the O.S.B. after the writer's name on the title page is traceable throughout the pages. The book is one of exceptional interest, and we commend it to all the reading public.

